# Genetic association studies for gene expressions: permutation-based mutual information in a comparison with standard ANOVA and as a novel approach for feature selection

**DOI:** 10.1186/1753-6561-1-s1-s9

**Published:** 2007-12-18

**Authors:** Silke Szymczak, Angelo Nuzzo, Christian Fuchsberger, Daniel F Schwarz, Andreas Ziegler, Riccardo Bellazzi, Bernd-Wolfgang Igl

**Affiliations:** 1 Institute of Medical Biometry and Statistics, University Hospital Schleswig-Holstein, Campus Lübeck, University at Lübeck, Ratzeburger Allee 160, 23538 Lübeck, Germany; 2 Department of Computer Science and Systems, University of Pavia, Via Ferrata 1, 27100 Pavia, Italy; 3 European Academy of Bolzano, Viale Druso 1, 39100 Bolzano, Italy

## Abstract

Mutual information (MI) is a robust nonparametric statistical approach for identifying associations between genotypes and gene expression levels. Using the data of Problem 1 provided for the Genetic Analysis Workshop 15, we first compared a quantitative MI (Tsalenko et al. 2006 J Bioinform Comput Biol 4:259–4) with the standard analysis of variance (ANOVA) and the nonparametric Kruskal-Wallis (KW) test. We then proposed a novel feature selection approach using MI in a classification scenario to address the small *n* - large *p *problem and compared it with a feature selection that relies on an asymptotic *χ*^2 ^ distribution. In both applications, we used a permutation-based approach for evaluating the significance of MI. Substantial discrepancies in significance were observed between MI, ANOVA, and KW that can be explained by different empirical distributions of the data. In contrast to ANOVA and KW, MI detects shifts in location when the data are non-normally distributed, skewed, or contaminated with outliers. ANOVA but not MI is often significant if one genotype with a small frequency had a remarkable difference in the average gene expression level relative to the other two genotypes. MI depends on genotype frequencies and cannot detect these differences. In the classification scenario, we show that our novel approach for feature selection identifies a smaller list of markers with higher accuracy compared to the standard method. In conclusion, permutation-based MI approaches provide reliable and flexible statistical frameworks which seem to be well suited for data that are non-normal, skewed, or have an otherwise peculiar distribution. They merit further methodological investigation.

## Background

Gene expression varies substantially in humans, and variation in the expression level of many genes is heritable. One of the most challenging aspects is, however, the identification of associations between genotypes and gene expression levels, for which transcript levels are viewed as phenotypes. This has become more important as high throughput technologies enable researchers to genotype single-nucleotide polymorphism (SNP) markers on a genome-wide scale and to measure gene expression levels of thousands of genes simultaneously. However, regardless of the final goal of a specific study, a number of problems arise frequently, related to the very nature of the genetic data. Herein, we have identified two such problems in which the use of standard statistical methods may be inappropriate or provide suboptimal results.

A first particular challenge is that gene expression data often are not normally distributed [1]. In many situations, data distributions are highly skewed, contaminated with outliers, or even completely unknown. Consequently, common parametric approaches like the standard analysis of variance (ANOVA) are often inadequate for statistical inference. The underlying model assumptions such as normality are substantially violated, and robust nonparametric methods are reasonable alternatives.

A second challenge is caused by the small *n* - large *p *problem because a relatively small number of subjects is classified by using a relatively large number of genotypes. Subsequently, an almost infinite number of possible solutions explain the data equally well, and a key problem is to reduce the number of attributes using a suitable feature selection strategy.

One approach to tackle the two problems is the use of mutual information (MI), a nonparametric approach from information theory. First applications of its use for analyzing genotypes and expression data have been published very recently [2,3]. In particular, Tsalenko et al. [4] proposed a quantitative MI score (QMIS) for analyzing SNP-expression association matrices.

First, we compare the permutation-based QMIS of Tsalenko et al. [4] with the standard statistical approaches in an ANOVA-type setting. Specifically, we systematically investigate the causes for differences in statistical test results of QMIS, the standard parametric ANOVA, and the Kruskal-Wallis (KW) test. We show that these can be explained by the distributions of gene expression levels conditional on the SNP genotypes.

In the second application, we aim at classifying the molecular phenotype data of subjects obtained from clustered gene expression profiles using a small set of features. In particular, we propose a novel permutation-based MI strategy for feature selection and compare it with a standard feature selection approach which relies on the asymptotic *χ*^2 ^distribution.

In both applications, we utilize the Genetic Analysis Workshop (GAW) 15 Problem 1 data.

## Methods

### Data

Data for Problem 1 of GAW 15 come from 14 three-generation CEPH (Centre d'Etude du Polymorphisme Humain) Utah families. All 194 members of these families were genotyped at 2882 autosomal and X-linked SNPs. Expression levels in lymphoblastoid cells were measured for 8500 genes. 3554 transcripts were selected by Morley and colleagues [5] because of greater variation among individuals than between replicated measurements. For all analyses, we used log-transformed expression levels.

### Mutual information

The concept of MI is based on the entropy of a random variable. Shannon [6] defined entropy as a measure of uncertainty in predicting a future value of a random variable *X*. In other words, the entropy can be viewed as a quantity representing the information associated with an outcome of *X*. In case of a discrete random variable *X *with outcomes *x *∈ X, the entropy is given by

$$H(X)=-\sum_{x\in X\;}p_{X}(x)\log_{2}p_{X}(x)=\sum_{x\in X\;}p_{X}(x)\log_{2}\frac{1}{p_{X}(x)},$$

where *p*_*X*_(*x*) = *P*_*X*_(*X *= *x*), and X denotes the sample space of *X*. The entropy is non-negative, disappears in case of a degenerate random variable and is maximal for a uniformly distributed *X*. For estimation, we use empirical frequencies ${\hat{p}}_{X}(x=i)=\frac{n_{i}}{n}$, where *n*_*i *_is the number of observations with *X *= *i*, and *n *is the total sample size. A natural estimator of *H*(*X*) is thus given by $\bar{H}(X)=-\sum_{i=1}^{\left|X\right|}\frac{n_{i}}{n}\log_{2}\frac{n_{i}}{n}$, where |X| is the cardinality of X.

MI extends the latter concept to random variables *X *and *Y*, and measures their mutual dependence. In the discrete case, *MI*(*X*, *Y*)

is defined as

$$MI(X,Y)=\sum_{x\in X}\sum_{y\in Y}p_{XY}(x,y)\log_{2}\frac{p_{XY}(x,y)}{p_{X}(x)p_{Y}(y)},$$

where *p*_*XY*_(*x*, *y*) denotes the joint probability of *X *and *Y*, and *p*_*X*_(*x*) and *p*_*Y*_(*y*) are the corresponding marginal probabilities. *MI*(*X*, *Y*) quantifies the amount of information that one random variable contains about the other. Equivalently, *MI*(*X*, *Y*) measures the reduction in uncertainty of *X *if *Y *is known, and vice versa. Thus, for two independent variables *X *and *Y*, observing one of them does not lead to an information gain concerning the other one. In this case, *p*_*XY*_(*x*, *y*) = *p*_*X*_(*x*)·*p*_*Y*_(*y*), so that the ratio equals one and, subsequently, *MI*(*X*, *Y*) = 0. The other extreme case occurs if *X *and *Y *are completely linearly related. Then, all information conveyed by *X *can be found within *Y*. *MI*(*X*,*Y*) can then be calculated using the entropy of one of both variables.

Moreover, *MI*(*X*, *Y*) is symmetric, non-negative and is related to the entropy via

*MI*(*X*, *Y*) = *H*(*X*) - *H*(*X *| *Y*) = *H*(*X*) + *H*(*Y*) - *H*(*X*, *Y*),

where *H*(*X *| *Y*) denotes the conditional entropy, and *H*(*X*, *Y*) is the joint entropy of *X *and *Y*. Because *H*(*X *| *X*) = 0, we obtain *H*(*X*) = *MI*(*X*, *X*), and *H*(*X *| *Y*) ≥ 0 yields *MI*(*X*, *X*) ≥ *MI*(*X*, *Y*). In other words, no other variable can contain more information about *X *than *X *itself.

For discrete random variables, the distribution of *MI*(*X*, *Y*) can be approximated by a second-order Taylor series. In case of stochastically independent random variables *X *and *Y*, the resulting expression is related to a *χ*^2^-test of independence and follows a gamma distribution [7]. As a consequence, optimal asymptotic statistical properties of the *χ*^2^-test also hold for MI [2]. However, the required regularity conditions are not always met. Thus, the approximation to the *χ*^2 ^distribution is imprecise if sample sizes are small, if the numbers per class differ substantially, or if the number of classes is small. In these cases, the use of empirical *p*-values as obtained from a permutation procedure is preferable.

If one of the variables, say *Y*, is continuous with observations *y *∈ ℝ, a QMIS can be calculated [4]. To this end, a threshold *q *is determined, leading to a dichotomous variable *Y*_*q *_such that *Y*_*q *_= *I*(*Y *<*q*), where *I*(·) denotes the indicator function. *QMIS*(*X*, *Y*) is then defined as the maximum possible MI:

*QMIS*(*X*, *Y*) = max_*q*∈ℝ _*MI*(*X*, *Y*_*q*_).

Analogously to the entropy, relative frequencies can be used to determine $\bar{MI}(X,Y_{q})$. For a given threshold *q *and using the right hand side of eq. (1), an estimator is given by

$$\bar{MI}(X,Y_{q})=\sum_{i=1}^{\left|X\right|}\sum_{j=1}^{2}\frac{n_{ij}}{n}\log_{2}\frac{n_{ij}}{n}-\sum_{i=1}^{\left|X\right|}\frac{n_{i.}}{n}\log_{2}\frac{n_{i.}}{n}-\sum_{j=1}^{2}\frac{n_{.j}}{n}\log_{2}\frac{n_{.j}}{n},$$

where $n_{i.}=\sum_{j=1}^{2}n_{ij}$ and $n_{.j}=\sum_{i=1}^{\left|X\right|}n_{ij}$ and *n*_*ij *_is the number of observations in group *j *defined by *Y*_*q *_with *X *= *i*.

The estimator given in Eq. (3) is solely based on frequencies and does not involve any continuous measurements such as squared deviations from a mean value. As a consequence, the QMIS approach is robust against outliers and does not require any specific shape of distribution.

### Application 1: comparison of QMIS with ANOVA and KW

In the first application, we compared the permutation-based QMIS with the standard parametric ANOVA and the nonparametric KW for detecting associations between a single gene expression level and a single SNP. We systematically investigated the causes for differences in statistical test results. We calculated QMIS according to Eq. (2) using estimates obtained from Eq. (3). To specify a final threshold for each gene, the range of expression values was divided into 10 equidistant intervals, yielding 11 potential thresholds within the maximization process. The number of intervals was limited to reduce computation time.

Empirical *p*-values were computed using a permutation procedure. In doing so, for each of the *N *= 10^4 ^permutations, genotypes were fixed, while gene expressions were permuted. In cases where no permuted test statistic was at least as extreme as the test statistic using the original data, calculations were repeated with *N=*10^8 ^permutations. To allow for heteroscedastic errors, the Welch modification of the parametric ANOVA was used. Finally, we denoted an association of gene *i *and transcript *j *to be significant if the *p*-value is less than *α*_local _= 10^-4^.

To avoid within-family dependencies, we only used founder data for model comparisons. In addition, SNPs with less than 5% of samples for each genotype and more than 5% of missing genotypes were removed, yielding 3554 genes and 1089 SNPs.

Differences in *p*-values between the different approaches were visualized using hexagon binning [8]. All computations were done using the computer program R including special parts implemented in C.

### Application 2: feature selection in classification

In the second application, we aimed at classifying the molecular phenotype data using a small set of features.

Within the first step, we created clusters of individuals with similar gene expression profiles. The data were modeled as a finite mixture of Gaussian distributions, and the most likely cluster partition was determined by an expectation-maximization (EM) algorithm using a 10-fold cross-validation procedure to determine the optimal number of clusters. After the clustering step we assigned a label to each individual, which was used to perform a supervised learning task on the basis of the genotype information.

**Figure 1:**
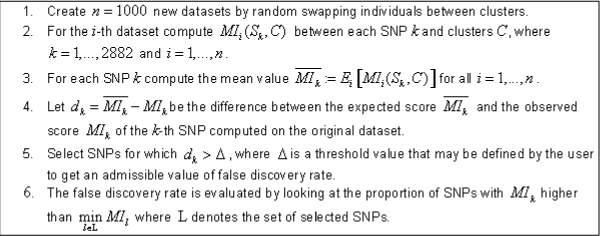
Feature selection based on permutation method.

In what followed, we considered SNPs and clusters as random variables and computed the MI between them. We evaluated the significance of MI in two ways. First, we focused on a parametric approach and used the relation between MI and the *χ*^2^-test of independence. More precisely, we considered a *χ*^2^-test in which the degrees of freedom (d.f.) are equal to (number of genotypes - 1)·(number of classes - 1) at a significance level of *α *= 0.001. Second, we applied a novel nonparametric approach based on a permutation method inspired by the significance analysis of microarrays (SAM) technique [9]. This approach allowed us to evaluate the expected false discovery rate (FDR) on the empirical "null" distribution of MI values by choosing a threshold for the MI. Conversely, by defining a desired FDR, we could choose a suitable corresponding threshold value for MI (Figure 1).

The step following feature selection was classification. We estimated the goodness-of-fit of the model using a 10-fold cross-validation. We determined the accuracy of the classification procedure as the proportion of correctly classified individuals. A person was classified correctly if the classification step led to that class that was specified in the previous cluster analysis. In general, a cross-validation resolves the problem of obtaining a test set, but it is affected by a selection bias that is often ignored. Here, the selection bias may result from performing feature selection on the entire data set before running a cross-validation. In this way, all the cross-validation learning steps are affected by the bias provided by feature selection, because the entire data set was already used for selecting the most interesting features. To overcome this problem, we included the feature selection step in the cross-validation procedure, as recommended by Simon et al. [10], to select the most informative SNPs on each of the 10 training sets and to use them only on the corresponding *j*^th ^test set.

Because feature selection was made in each iteration step of the cross-validation, a different SNP list was generated in each run of the procedure. To derive a consensus SNP list, we extracted those SNPs that were selected in each run of the cross-validation process. Therefore, this set contained the most important SNPs regardless of small changes in the training set.

The WEKA software version 3.4 [11] was employed for both clustering with 10-fold cross-validation and classification using the learning machines naïve Bayes, support vector machine (SVM) and *k*-nearest-neighbor (KNN). For the number of neighbors in KNN, we chose *k *= 1, 5, 10, and for all learning machines we used the default settings of the package.

## Results and discussion

### Application 1: comparison of QMIS with ANOVA and KW

**Figure 2:**
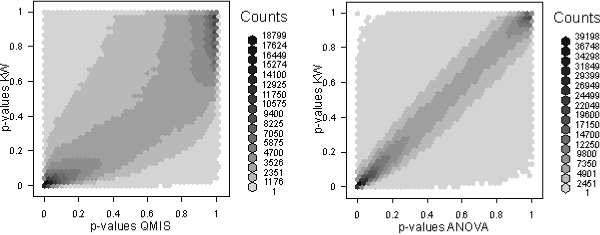
**Scatterplots of *p*-values using a hexagon binning procedure**. Left, QMIS and KW. Right, ANOVA and KW.

The three statistical methods QMIS, ANOVA and KW produced remarkably different results. First, the total number of significant associations varies between 117 for the KW, 340 for QMIS, and 3149 for ANOVA. Only 28 associations are significant in all three approaches. In other words, concordant results are less frequent than model-specific significances. When looking at all *p*-values, substantial discrepancies between QMIS and the KW become apparent (Figure 2, left), whereas *p*-values from ANOVA and the rank-based ANOVA-type KW are more comparable (Figure 2, right).

**Figure 3:**
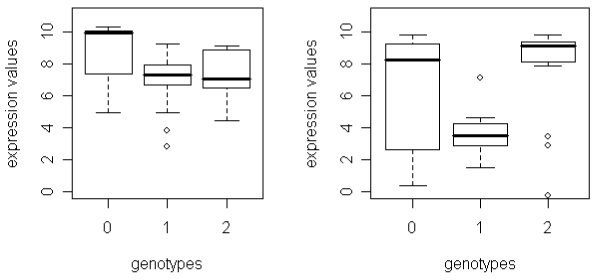
**Gene expression levels by genotypes in cases with divergent results for QMIS, ANOVA, and KW**. Left, QMIS significant, ANOVA not significant. Right, QMIS significant, KW not significant. Box plots display median, quartiles, largest non-outlier and extremes.

We now give an explanation for these discrepancies and, for this purpose, consider situations in which only one statistical procedure led to a significant result. The left plot in Figure 3 illustrates a scenario in which QMIS is significant, but ANOVA is not. Here, substantially different data distributions are observed for the three genotypes. More specifically, the first is left-skewed, the second is symmetric, and the third is right-skewed. Obviously, these groups are quite similar in variability but differ in location. However, mean values are inadequate for estimating the individual location parameters because of the different shapes of the empirical distributions and should therefore not be used for a statistical comparison with a global center. As a consequence, the parametric ANOVA procedure does not detect the obvious differences in location. In contrast, QMIS is solely based on frequencies and does not involve real valued distances. Thus, this mechanism is robust against variation in skewness and also robust against outliers. In such a scenario, the QMIS procedure is able to detect differences in location.

In cases where ANOVA is significant but QMIS is not, we often observed one genotype with a small frequency which, at the same time, showed a remarkable difference in the average gene expression level to the other two genotype groups. QMIS cannot detect these differences because it depends heavily on genotype frequencies.

The right plot of Figure 3 gives an example for a significant *p*-value from QMIS but a non-significant KW. Again, distributions are dissimilar in shape and differ in both variability and skewness. The KW assumes, however, similar types of distributions per group to analyze shift alternatives. Consequently, only the frequency-based QMIS approach is able to detect the apparent differences in location.

Interestingly, whenever the *p*-value of the KW is significant, the *p*-value from QMIS is small. Thus, QMIS seems to be more sensitive to any kind of difference in gene expression levels between genotype groups. In principle, a MI strategy without dichotomizing the expression level might be superior. Such a procedure uses the complete information of a continuous variable and was proposed by Dawy et al. [2]. It is, however, computationally intractable because of the required numerical integration for association-expression matrices. Furthermore, results can be unstable [2].

Finally, all three approaches, i.e., QMIS, ANOVA, and KW are significant only in situations in which there are extreme differences in location parameters.

### Application 2: feature selection in classification

In an initial step, five clusters were identified by the EM algorithm. Then classification was performed using three different classifiers with both feature selection criteria described above, showing that the nonparametric approach improves classification performances in contrast to the parametric one. Using the *χ*^2^-test with 8 d.f., more than 350 SNPs were selected at each iteration of the cross-validation, yielding a best final accuracy of about 50%. In contrast, feature selection based on MI with permutation reduces the number of informative SNPs to 200 with a final accuracy of 64.66% using naïve Bayes classifier. SVM and optimal KNN (*k *= 1) have slightly smaller classification accuracies of 62.37% and 54.65%.

Seventy-five SNPs are always selected among the different training-test pairs and therefore comprise the final SNP list. If only these SNPs are used for classification, the accuracy of the naïve Bayes classifier increases to about 70%. This is in line with previous observations using random forests as classifier [12].

## Conclusion

A permutation-based QMIS is a sensible nonparametric alternative to the standard parametric ANOVA and the nonparametric KW. In contrast to ANOVA and KW, QMIS is able to detect differences in location between asymmetric, skewed, and outlier-contaminated distributions. However, parametric methods are generally more powerful if model assumptions such as normality are met. Similarly, KW might be more adequate than QMIS for analyzing a shift alternative in case of equally shaped distributions. However, these assumptions are not met for gene expression data in the majority of cases.

In addition, our novel permutation MI strategy for feature selection in a classification setting has higher accuracy and uses a smaller number of SNPs than the standard *χ*^2 ^approach.

## Competing interests

The author(s) declare that they have no competing interests.
